# The Cutaneous Physiological Redox: Essential to Maintain but Difficult to Define

**DOI:** 10.3390/antiox9100942

**Published:** 2020-10-01

**Authors:** Sapir Ron-Doitch, Ron Kohen

**Affiliations:** Institute for Drug Research, School of Pharmacy, Faculty of Medicine, Hebrew University of Jerusalem, Jerusalem 9112000, Israel; sapirr@ekmd.huji.ac.il

**Keywords:** skin redox, physiological redox, Nrf2–Keap1, cutaneous ALEs, skin microbiome

## Abstract

Skin is a unique tissue, possessing extremely efficient protective and regulative mechanisms, similar only to the gut and lungs. These tissues serve as an interface with the environment and are exposed to stressors from both endogenous and exogenous sources. Interestingly, all these stressors lead downstream to a cellular production of reactive oxygen species (ROS) and other electrophiles, which, in turn could have deleterious outcomes for the living organism. Hence, such tissues should always maintain a “high-alert” condition in order to cope with these various insults. Nevertheless, a moderate production of ROS induced by stressors could actually be beneficial, although it is impossible to predict if and which exposure would lead to which outcome. Consequently, a parameter which would indicate the skin’s readiness to cope with continuously fluctuating conditions is required. It has been proposed that the redox status may serve as a suitable indicator. In this opinion manuscript, we argue that the redox status is a vague parameter that is difficult to characterized and quantify due to its extremely dynamic nature. The common convention that the redox status is composed solely of the balance between oxidants and reductants (ROS and antioxidants) is also thought-provoking. Since this parameter in vivo behaves in a dynamic and complex manner, it better fits the description of a process, rather than an individual parameter. We suggest that the homeostatic modulation of the physiological redox (PR) should be in focus, rather than the redox status parameter itself. It is further suggested that low molecular weight antioxidants (LMWA) are, in fact, rather insignificant concerning the PR maintenance, and that the major contributors to this delicate modulation are regulative, protein-based systems such as the protective phase II antioxidant enzymes. Moreover, we show that skin microbiome and cutaneous advanced lipid peroxidation end-products (ALEs) take part in sustaining the cutaneous PR homoeostasis via activation of the Nrf2–Keap1 protective pathway.

## 1. Introduction

Oxidative processes accompany the living organism from birth to death, being the driving force of cellular progression through the natural life cycle [1,2]. Oxidative reactions play a role in numerous biochemical events and pathways, which are crucial for many normal cellular functions such as aerobic metabolism, immune system activity and various signal transduction pathways [1,3,4,5,6]. The concept of “oxidative stress” (OS) is widely used to describe an excessive oxidant challenge and changes in the cellular redox equilibrium. Nevertheless, living cells are constantly under some extent of oxidative stress. While this is an ordinary physiological condition, an excess of oxidative stress would be deleterious [7]. The involvement of oxidation reactions in almost every function of the cell leads to the use of many descriptive and generalizing terminology in the scientific literature, such as stress, oxidative stress, eustress, redox and related terms [8,9,10]. Some of these expressions share similar meanings, though they are often being used in an incorrect context. Specifically, for example, there is a tight connection between the more general concept of stress and oxidative stress phenomenon, since in most cases the first induces the latter. The delicate balance between the two aspects of oxidation, also known to as stress and eustress, is referred to as redox status, redox homeostasis or redox equilibrium. However, unlike many biochemical parameters which have a fixed value or a physiological range (e.g., pH) [10], it is practically impossible to determine a definite baseline and even to successfully distinguish between these parameters in a cell, tissue or the entire organism. Indeed, there is much debate in recent years regarding redox status definition and characterization [9]. In order to define this parameter there is a need for suitable biomarkers and appropriate methods [11]. However, many unsuccessful attempts have been made to find such physiological biomarkers for quantification of OS, stress, eustress and their interactions. In fact, no universal criteria for these parameters are acceptable among most researchers [12].

In this opinion manuscript, we relate to the skin as the most suitable organ to examine and address these parameters, as well as to investigate the effects and consequences of stress. We propose that the difficulties in evaluating redox status in the skin arise from its dynamic, ever-changing characteristics and the continuous exposures of the skin to numerous endogenous and exogenous insult sources. We also suggest the importance of the skin microbiome and endogenous lipid peroxidation processes as mediators in maintaining the delicate cutaneous redox balance.

## 2. Skin: A Suitable Tissue to Address Redox Homeostasis

Our skin—an essential part of the integumentary system—is our interface with the external environment. It serves as a protective physical and biologic barrier, shielding our delicate internal surroundings from various insults such as air pollutants [13], ionizing and nonionizing radiation, toxins, wounds and microbial invasions [14,15]. The human skin is also a lush microbial habitat, hosting a wide variety of bacteria, fungi and viral species [16]. The continuous exposure of skin to exogenous and endogenous insults drove it to develop powerful defense mechanisms which are always ready to efficiently respond to various insults, similarly to the respiratory and gastrointestinal tracts. Therefore, it is abundantly clear why these tissues must maintain highly sensitive stress-sensing and response mechanisms. One such mechanism is the cutaneous redox homeostasis, which is very carefully monitored and maintained [17]. Impairments in the delicate redox homeostasis of the skin may have many deleterious effects, including premature aging, pigmentation and malignancies [1,18,19,20,21,22].

## 3. Stress Definition, Cutaneous Stress Terminology and Terminological Debate

The debate concerning the often-used terms OS, eustress and stress (distress) is far from settled [3,8,9,10,14,23,24]. Since endocrinologist Hans Selye proposed the use of the terms distress and eustress in the 1970s [25], they have been used extensively in various fields, including clinical health, psychology, physiology and pathophysiology—as well as to describe the effects of environmental factors on the human body [23,24,26]. Many times, however, authors fail to clearly distinct between beneficial stress and deleterious stress, i.e., eustress and distress. The scientific literature is flooded with manuscripts dealing with these topics, while in many cases the terminology is incorrect or unfitting, so much so that it sometimes leads to faulty interpretations of the obtained research results. Moreover, the fact that there are no reliable means to measure and quantitate OS and define its type and severity, adds to the confusion in this scientific field [12]. It is apparent, that OS in its traditional definition as a global imbalance of pro-oxidants and antioxidants is neither accurate nor sufficient. Dean J. suggested that a more suitable definition of OS is “a condition that disrupts redox signaling and control” [27]. The oxidative status in cells—often mistakenly termed “redox”—is considered to be slightly oxidative, meaning the pro/antioxidant scale leans towards the oxidants [14]. Clearly, as a pivotal part of cellular physiology, the redox state must be carefully monitored and controlled in a feedback-based manner. Dean P. Jones and Helmut Sies [28] referred to the phrase “the redox code” as a set of principles that defines the positioning of cellular redox-related mechanisms in space and time in biologic systems. This description seems very accurate, as it considers the complexity and delicacy of the cellular endeavors to modulate its redox status [4]. There are several regulative mechanisms which can affect the redox status and its hemostasis in the cell/tissue, such as the thiol/disulfide system and the nuclear factor erythroid 2-related factor 2–Kelch-like ECH-associated protein 1 (Nrf2–Keap1) pathway (see below) [29,30,31]. The term “redox status” aims to describe the relationship between OS and its counterparts in a normal, baseline setting. However, it is impossible to refer to a “natural” or “normal” cutaneous redox status or redox equilibrium. Since the skin is constantly exposed to exogenous and endogenous insults that challenge its homeostasis and affecting many physiological parameters, it does not have a “normal” resting state which can be evaluated and considered as a baseline. In fact, the terms “redox” or “redox potential” are not at all suitable to be used in describing biologic environments [10]. Redox is a thermodynamic parameter, which can only be determined under thermodynamic conditions, while such conditions do not exist in biology in general and specifically in the skin. For example, the cutaneous tissue is never in a real equilibrium and the biochemical reactions occurring in this setting are not fully reversible. Therefore, in order to distinguish between the redox parameter in chemistry and in biology, we should refer to it as **physiological redox (PR) and its homeostasis**, in order to describe a dynamic redox status when referred to in biologic context.

**Figure 1:**
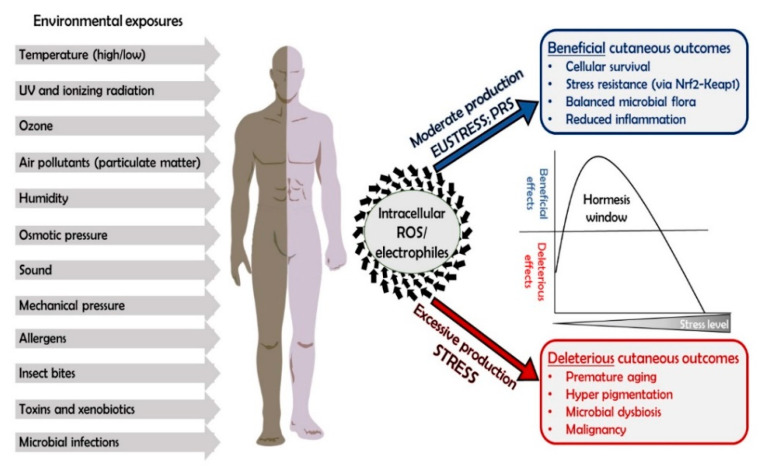
**Stress exposure is translated to “oxidative stress” (OS)**. Many exposures of the skin to the elements (either physical, chemical or biologic) ultimately leads to the augmentation of reactive oxygen species (ROS) intracellular concentrations. This may have either deleterious or positive outcomes for the skin, depending on the duration and location of the exposure and on the downstream cellular response (i.e., ROS induction levels). This phenomenon sustains the principal of hormesis, meaning that a moderate production of ROS leads to the maintenance of a healthy PR, while excessive or insufficient ROS levels lead to detrimental cutaneous consequences.

The difficulty of accurately evaluating and describing OS and PR and its homeostasis is strongly manifested in the skin, which is a large and nonhomogenous tissue, in which the physiological and biochemical condition greatly differs between one site to another. Thus, stress can transform into eustress and vice versa, depending on the specific location, physiological and environmental conditions at the time of measurement, as well as other related factors (for example, “fight or flight” response). The relationship between these two definitions (stress and eustress) is constantly shifting, and therefore, in the context of skin and its highly dynamic nature, the term oxidative stress, or simply stress, is more appropriate than the differentiation into eustress and stress. This is emphasized by the interesting observation that many stress exposures of the skin lead to a production of intracellular electrophiles and ROS and may be either beneficial or deleterious (Figure 1). Therefore, stress always leads to oxidative stress, and so the expression “stress” by itself is sufficient. Due to its continuous exposures, the skin is equipped with sensitive regulation mechanisms which are responsible for the removal of an excess of deleterious oxidants and regenerating reducing equivalents, thus maintaining the PR in a dynamic equilibrium [1,8].

## 4. Cutaneous PR Homeostasis—A Hormetic Mechanism

One of the pivotal oxidation regulation mechanism in the skin is the Nrf2–Keap1 pathway, and its activity leads to PR maintenance which evidently sustains the principal of hormesis. According to Mark P. Mattson [32] hormesis is defined as: “a process in which exposure to a low dose of a chemical agent or environmental factor that is damaging at higher doses induces an adaptive beneficial effect on the cell or organism”. Indeed, this concept perfectly describes the ever-changing cutaneous PR homeostasis, in which a low dose of an oxidative driving force is required and beneficial to the cells, yet high doses of oxidative stress are toxic, leading to cellular damage and death (Figure 1) [33]. Therefore, it is the level of electrophiles and ROS produced intracellularly following stress exposure (or extracellular oxidants that will penetrate the cells and elicit response) that will determine whether the outcome of the stress would be deleterious or beneficial. Evidently, skin homeostasis of the PR is a dynamic state, and therefore requires an inducible, highly reactive and modifiable control system for maintaining a healthy, mildly oxidative status, in order to overcome excessive oxidative insults [14]. The skin is equipped with a well-developed antioxidant defense system, in charge of maintaining a “healthy PR”. It is traditionally divided into two branches: low molecular weight antioxidants (LMWA) and the antioxidant enzymes. We argue here, that the LMWA do not significantly contribute to PR maintenance and that alternatively, most of its control and modulation can be attributed to the antioxidant enzymes.

## 5. Do LMWA Significantly Affect PR Homoeostasis?

The notion that PR is a parameter under delicate control indicates that its components should respond to small changes in their concentration. Therefore, LMWA, which are considered as one of the major components contributing to this parameter, should respond quickly to small changes in PR values and in its delicate homeostasis. In other words, the cells should be able to recruit such compounds or to synthesize and regenerate them in a short period of time. However, this is not the case for this group of compounds. The direct-acting antioxidants, the scavengers, are all small molecules abundantly found in the living organism tissues [14,34]. They share a similar chemical trait: the ability to donate electrons to oxygen metabolites, i.e., scavenging the radical, thus preventing it from attacking biological targets [1,34]. Therefore, all scavengers are considered as reducing agents and as such are believed to be major contributors to PR maintenance. The group of reducing antioxidants (scavengers) contains lipophilic and hydrophilic compounds and possess many advantages such good cellular penetration and wide spectrum of activity. Two major factors determine their ability to act as protective antioxidants: their concentration and the rate constant of their reaction with the oxidant [10,14]. Of note, is that any scavenging action of LMWA requires very close proximity to the ROS microenvironment as well as high enough concentrations to ensure efficient detoxification and neutralization [1,8,14]. Statistically, however, it is very unlikely that an electrophile (ROS) would encounter a scavenger in its vicinity before it would be able to interact with the surrounding biological macromolecules that are found in cells in extremely high concentrations and density [10]. Nevertheless, under certain circumstances, significant differences in rate constants can be compensated by differences in concentrations, and vice versa. Even non-radical oxidants such as hydrogen peroxide, which have a relatively long half-life, will most likely be decomposed by high-affinity enzymes (e.g., peroxidase, catalase) prior to a possible interaction with a scavenger. Moreover, a high-yield recycling mechanism of the LMWA is also needed for effective and consistent antioxidant activity and affecting PR homeostasis. Nonetheless, such recycling processes are rare, require the presence of enzymes and are “expensive” to the cell. For example, to regenerate ascorbic acid from its oxidized form—dehydroascorbic acid—two molecules of GSH are required, resulting in a depletion of the cellular pool of reducing equivalents [35]. There is also a lack of a regulatory mechanism for LMWA that maintains their concentrations constant enough to affect the PR homeostasis. In addition, although they possess a wide spectrum of activity toward a large variety of oxidants, only a minority of the reducing equivalents in our body actually originate from endogenous sources. It is quite surprising that the number of LMWA synthesized by our cells or generated as waste products [36,37] (and act as scavengers) is so limited. Seemingly, there is not even one compound synthesized in humans specifically to fulfill solely an antioxidant role. Even GSH—which is produced in the cells in high concentrations—possesses many biochemical roles besides being an antioxidant [1,38]. Although it is considered a reducing agent, GSH cannot donate its electron in vivo directly (such as in the case of ascorbic acid, for example), but rather requires the mediation of an enzyme (e.g., glutathione-S-transferase, GST). Other reducing compounds such as NAD(P)H, also requires the involvement of an enzyme for their antioxidative action [1]. It is therefore questioned whether LMWA alone possess a physiological relevance in modulating the delicate cutaneous PR homeostasis. It is suggested that another, more sophisticated and tightly regulated system, governs the skin PR maintenance processes, as previously discussed [33,39].

## 6. The Importance of the Antioxidant Enzymes in Maintaining PR Homeostasis

In contrast to LMWA, the antioxidant enzymes are controlled by a tightly regulated system. These enzymes are part of the larger group of cytoprotective phase II enzymes and are responsible for the maintenance of a healthy PR within the cells. These include superoxide dismutase (SOD), catalase, peroxidase, NAD(P)H dehydrogenase [quinone] (NQO1), heme oxygenase 1 (HO-1), gluthathione reductase (GSR) and more [14]. Commonly, these enzymes possess high reaction rates and highly efficient specific activities, e.g., catalyzing ROS-scavenging reactions, recycling and regeneration of LMWA, synthesis and catalysis of GSH associated reactions, etc. For instance, HO-1 catalyzes the degradation of heme into carbon monoxide, iron and biliverdin which can be further converted to bilirubin, a nonenzymatic antioxidant [37,40] This diverse family of enzymes are also considered to be a primary defense mechanism against many degenerative and chronic disease conditions [41,42]. Another group of important structural proteins which also possess antioxidant properties in the skin are the small proline-rich proteins (SPRRs), which are cross-bridging proteins in squamous epithelia and also possess the ability to quench ROS by forming intramolecular disulfide bonds [43]. In contrast to LMWA, these antioxidant enzymes are part of a tightly regulated and inducible, feedback-based system. The key regulator mechanism which governs these enzymes’ activities is the Nrf2–Keap1 pathway [17,44].

## 7. Skin- and Bacteria-Derived Metabolites Affect PR Homeostasis via Activation of the Nrf2–Keap1 Pathway

### 7.1. Nrf2–Keap1 System

Nrf2 is a transcription factor that is found in the cytoplasm and under normal conditions is bound to two molecules of the cysteine-rich metalloprotein Keap1. Normally, Keap1 mediates Nrf2 proteosomal degradation via ubiquitination, resulting in Nrf2 cytoplasmic half-life of approximately 10–20 min [29,45]. Upon induction of oxidative stress (electrophilic stress), an electrophile recognizes specific cysteine residues on the Keap1 protein and oxidizes them, resulting in the suppression of Keap1-mediated proteasomal degradation of Nrf2, thereby leading to its stabilization, translocation and accumulation within the nucleus [46]. In the nucleus, Nrf2 can undergo heterodimerization with various proteins such as small Maf proteins, followed by its binding to the antioxidant response element (ARE), also referred to as electrophile response element (EpRE) in the regulatory sequence of its target genes [47,48]. The result of these events is the induction of large networks of genes encoding enzymes and detoxifying proteins, including antioxidant enzymes and anti-inflammatory cytoprotective proteins [47,48,49]. The Nrf2–Keap1 pathway can be induced in two general manners, direct or indirect. Direct Nrf2 activation [50] occurs when an electrophilic entity enters or accumulates in the cytoplasm. Such molecules may be ROS, oxidative xenobiotics, nutrients, etc., or endogenous electrophiles deriving from cellular metabolism and lipid peroxidation processes occurring, for instance, in the skin and/or cutaneous bacteria [51,52,53]. The indirect activation mechanism includes intracellular production of endogenous ROS in low concentration in response to certain stress stimuli (e.g., UV irradiation, air pollutants, mosquito bites, etc.), which in turn induces the pathway. This system, particularly in the skin, should always be in alert in order to cope with stress conditions. This requires a continuous efflux of stimuli for this pathway in the skin. Surprisingly however, we have yet to encounter reports of natural, skin-derived Nrf2–Keap1 pathway activators in current literature (with the exception of α,β-unsaturated aldehydes). It is highly striking that such a pivotal mechanism will have no endogenous triggers. We have very recently proposed [51] two possible skin-associated activators for this important pathway, lipid peroxidation end products and the cutaneous microbiome.

### 7.2. Naturally Occurring Cutaneous Nrf2–Keap1 Activators—Lipid Peroxidation end Products

**Figure 2:**
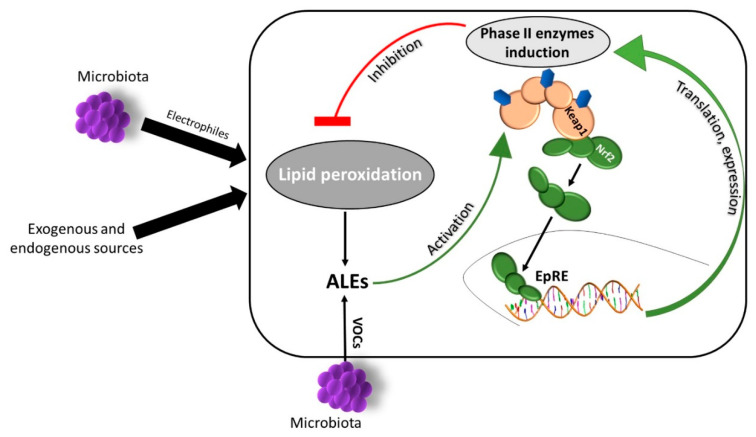
**Cutaneous lipid peroxidation processes are modulated in a feedback based manner mediated by the Nrf2–Keap1 pathway**. Physiological lipid peroxidation is naturally occurring in the skin in response to oxidative stimuli by environmental factor as well as by microbiota excretions and emanations, as well as direct interactions. Advanced lipid peroxidation end-products (ALEs are the end products of these processes but can also be contributed by resident cutaneous bacteria in the form of volatile organic compounds (VOCs). These compound can in turn activate the nuclear factor erythroid 2-related factor 2–Kelch-like ECH-associated protein 1 (Nrf2–Keap1) pathway by inducing Keap1 cysteine residues oxidation, leading to the expression of cytoprotective phase II enzymes. Then, these enzymes act in a negative feedback, inhibiting excessive lipid peroxidation, thus contributing to a healthy and balanced skin physiological redox (PR) homeostasis.

Hundreds of volatile organic compounds (VOCs) and odorous molecules are emitted from the human body, emanating from exhaled breath, urine, feces, saliva and skin [54]. The production of VOCs by cutaneous tissue is governed mainly by the secretion of three types of glands: eccrine, sebaceous and apocrine [55,56]. The skin microbiota also contributes greatly to human odor production, since microbes are most abundant in the vicinity of skin glands, where they metabolize fatty acids and their secretions [57]. VOCs emanating from human skin are mostly carboxylic acids and derivative esters, aldehydes, alkanes, short chain alcohols and ketones, which are generally advanced lipid peroxidation end-products (ALEs). Production of these compounds is not unique to cutaneous bacteria, as it is also the result of lipid peroxidation in the skin cells. Cutaneous lipid peroxidation processes are initiated by ROS that react with sebaceous and epidermal lipids and are further propagated in the presence of oxygen—leading to the accumulation of ALEs [58]. The origins of radical initiators are oxidative environmental exposures and possibly also naturally occurring cutaneous bacteria which are a newly suggested source of oxidative entities (see below). Lipid peroxidation is a physiological process occurring naturally in the skin, but is nonetheless a chain reaction which can be deleterious to the cells and is often difficult to control, while its end products may interfere with normal cellular functions, affecting the delicate cutaneous PR homeostasis [59]. It is well established that α,β-unsaturated aldehydes (which are also ALEs) are able to activate the Nrf2–Keap1 pathway by direct Keap1 oxidation [52,60]. We suggest that other skin-derived ALEs may in turn activate the Nrf2–Keap1 pathway, leading to the induction of antioxidant enzymes, hence diminishing further peroxidation processes. Thus, the ALEs produced during the peroxidation process may actually activate a negative feedback mechanism. Indeed, our recently published work [51] shows that the cutaneous ALEs nonanal, decanal and benzaldehyde, induced the Nrf2–Keap1 pathway in human keratinocytes while also granting protection against UVB-induced apoptosis. Therefore, it can be speculated that there is a feedback-based mechanism which relies on a moderate induction of lipid peroxidation processes by ROS originating from cutaneous exposures, leading to the accumulation of ALEs. In turn, these compounds evoke skin protection via the activation of the Nrf2–Keap1 pathway, thus maintaining a balanced PR, contributing to its homeostasis (Figure 2).

### 7.3. Naturally Occurring Nrf2–Keap1 Activators—Cutaneous Microbiota

As suggested above, the PR regulation mechanism governed by Nrf2–Keap1 pathway should be constitutively active in order to maintain a healthy tissue. This can be achieved only by a continuous excitatory stimulus of the pathway. We suggested that apart from skin-derived ALEs, such stimulus may also be donated by skin microbiome. The human skin is home to a milieu of microorganisms, namely bacteria and fungi. These microbes are distributed among three ecological niches: dry, moist and sebaceous areas, in which the communities are mostly stable and conserved within the same individual [61,62]. Two traditional functions are commonly attributed to cutaneous colonizers, similarly to those of the gastrointestinal (GI) and respiratory tracts: competitive exclusion of pathogens and immune-modulating roles [63]. However, with the advancement of interdisciplinary research approaches, the importance of the human microbiome in the cross-talk between remote tissues as well as its influence on various organ systems have been emphasized. First, the phrase “gut–brain axis” was coined, followed by advanced derivatives such as the above mentioned “gut–brain–skin axis” and “microbiome–skin–brain axis” [64,65,66]. The gut–brain axis refers to a link between mental conditions (i.e., depression, anxiety, etc.) and the microbial composition and activity in the GI tract and vice versa, with regard to dysbiosis, increased inflammation and impaired barrier function of the GI lining [64,67]. More recent research also linked microbiome alterations to neurological disorders such as autism [68] and Alzheimer’s disease [69]. The gut–brain–skin axis was hypothesized some 70 years ago by dermatologists Stokes and Pillsbury, who proposed a mechanism of a GI link between mental conditions and skin disorders such as acne [70]. The term was later coined and discussed further [67,71], while the molecular basis for this interesting relationship may most likely be attributed to stress-related neurotransmitters [72]. Most recently, Hadian et al. [65] referred to the microbiome–skin–brain axis in the context of bacterial involvement in wound healing and it is psychological and mental effects. Clearly, the skin and its associated microbiome is highly involved in many systemic physiological and pathologic processes and its importance in overall health and disease becomes apparent, similarly to that of the gut microbiome. Evidently, it has been shown that commensal *Lactobacillus* spp. in the GI tract were able to induce the Nrf2–Keap1 pathway in epithelial cells via beneficial OS induction, or more accurately, by oxidative eustress induction [73]. We recently showed that a newly described cutaneous bacteria metabolite, 3-furaldehyde, induces the pathway in human keratinocytes [51], while also providing protection against UVB-induced apoptosis. We further suggest that microbial presence on the skin’s surface is sensed as a sort of moderate stress, which results, as in the case of other stressors, in endogenous production of electrophiles which in turn activate the protective Nrf2–Keap1 pathway. It is therefore apparent, that the skin does occupy natural inducers for its important, cytoprotective, PRH maintaining mechanism, Nrf2–Keap1. These can be endogenous ALEs, which may employ a negative feedback based mechanism of activation, as well as exogenous, but tightly associated cutaneous bacteria and their metabolites.

## 8. Conclusions

In summary, in this opinion manuscript, it is argued that exposure to a variety of stressors is fundamentally translated into oxidative stress due to downstream production of reactive oxidative entities, which can be either beneficial or deleterious to cells and tissues. It is impossible to predict whether a certain insult would be sensed as “eustress” or instead be toxic. This would depend on the tissue, the specific location within the tissue, the duration of exposure, its type and the counterparts present in the tissue at the time of exposure. The lack of appropriate and reliable methods to evaluate stress intensity, available defense mechanisms and cutaneous consequences, may sometimes lead to mistaken conclusions and assumptions among the scientific community. We also suggest that the skin— due to continuous cutaneous exposure to insults from exogenous and endogenous sources—is the most suitable tissue to study stress and its consequences—as well as PR homeostasis and its maintenance and modulation. Moreover, the delicate and dynamic cutaneous PR homeostasis cannot be quantified since there is no basal, stress-free state for the skin. PR is not a singular measurable parameter, but rather a complex biologic process that is constantly fluctuating. Nonetheless, this ever-changing state can be modulated and controlled by the Nrf2–Keap1 protective system that is highly sensitive to small changes in the level of stress and the resulted endogenous ROS accumulation. This pathway, along with the thiol/disulfide system, contribute to the maintenance of a healthy dynamic PR and its homeostasis, arguably much more efficiently than LMWA scavenger molecules. Finally, it is suggested that skin and bacteria derived ALEs, as well as direct skin–microbiota interactions, serve as natural constitutive moderate Nrf2–Keap1 pathway activators, thus contributing to PR homeostasis regulation.

## Abbreviations

ROS	Reactive oxygen species
PR	Physiological redox
OS	Oxidative stress
LMWA	Low molecular weight antioxidants
ALEs	Advanced lipid peroxidation end products
Nrf2–Keap1	Nuclear factor erythroid 2-related factor 2–Kelch-like ECH-associated protein 1
GSH	Gluthathione
SOD	Superoxide dismutase
NQO1	NAD(P)H dehydrogenase [quinone]
HO-1	Heme oxygenase 1
GSR	Gluthathione reductase
GST	Gluthathione S-transferase
ARE	Antioxidant response element
EpRE	Electrophile response element
VOCs	Volatile organic compounds
GI tract	Gastrointestinal tract
